# Economic empowerment and intimate partner violence: a secondary data analysis of the cross-sectional Demographic Health Surveys in Sub-Saharan Africa

**DOI:** 10.1186/s12905-021-01363-9

**Published:** 2021-06-12

**Authors:** Heidi Stöckl, Anushé Hassan, Meghna Ranganathan, Abigail M. Hatcher

**Affiliations:** 1 LSHTM, London, UK; 2 University of North Carolina, Chapel Hill, NC USA; 3 Faculty of Health Sciences, University of Witwatersrand, Johannesburg, South Africa; 4 Institute for Medical Information Processing, Biometry, and Epidemiology, Ludwig-Maximilians-Universität München, Munich, Germany

**Keywords:** Intimate partner violence, Economic empowerment, Sub-saharan africa, Demographic and health surveys, Couples data

## Abstract

**Background:**

Intimate partner violence (IPV) has been recognized as a defining human rights, development and public health issue of our time. Economic empowerment is one of the most promising interventions to reduce IPV in sub-Saharan Africa, yet the evidence around economic factors that are key to ensure a reduction in IPV are still mixed. Furthermore, there is a lack of clarity on what kinds of economic empowerment works for which population group. This paper seeks a more nuanced understanding, by investigating whether the associations between indicators of economic empowerment and physical and/or sexual IPV are similar between the general population of women and among urban versus rural and young, or middle aged women versus older women.

**Methods:**

Using couples data from 25 DHS surveys across 15 countries (n = 70,993 women and men aged 15 and above at time of survey), we analyse how household wealth, men’s and women’s education and employment status, decision making on women’s income, differences in education and employment of women and their partners and women’s cash income are associated with physical and/or sexual IPV. We also provide sub-analyses for both urban and rural areas and for women aged, 15 to 24 25 to 34 and 35 to 49.

**Results:**

Across all surveys, 20% of women reported physical and/or sexual IPV in the last 12 months. On the one hand, our findings reinforced certain well-established patterns between women’s economic empowerment and IPV, with women’s and men’s higher levels of education and increased household wealth  associated with a decrease in IPV, and women’s employment, especially if only the woman worked, and women earning more than her partner associated with an increase in IPV. Most patterns did not differ across urban and rural settings and age groups, but notable differences emerged regarding household wealth, women’s and men’s employment in the last 12 months and relative employment and education.

**Conclusions:**

Factors relating to women’s economic empowerment are  vital in understanding and addressing IPV. Our analysis indicate however that future interventions need to consider the differing needs of urban and rural areas as well as be targeted to different age groups.

**Supplementary Information:**

The online version contains supplementary material available at 10.1186/s12905-021-01363-9.

## Background

Intimate partner violence (IPV) has been recognised as a defining human rights, development and global health issue of our time. IPV remains intractably high, despite its status as a United Nations Sustainable Development Goal (Target 5.2) and a policy priority for many governments [1]. Worldwide, an estimated one in three women has experienced physical and/or sexual IPV or non-partner sexual violence in their lifetime and every third murdered woman is killed by an intimate partner [2, 3]. The prevalence of IPV is particularly high in sub-Saharan Africa, where approximately 37% of women report experiencing IPV during their lifetime [2].

Key advances in research have shown that IPV is a probabilistic event that is influenced by interacting factors operating across all levels of the social ecology [4]. However, major enduring questions remain unanswered, particularly around economic empowerment. For example, little is known about how relative resources, for example, differences in education, employment status or income in the household impact on violence. Moreover, literature around women’s employment and microcredit offer conflicting evidence—evaluations of such programmes at times show protective elements against partner violence and at times suggest increased risk [5–7]. The prevalence of IPV varies across age groups and between urban and rural areas, even within the same country. Programmes that have been tested and have shown to be effective in rural areas have often not shown the same effects in urban areas [8, 9]. Despite acknowledging these differences, researchers have rarely investigated whether economic empowerment affects these groups differently with respect to IPV.

Poverty, unemployment, lack of economic opportunity and gender inequalities are structural factors that shape women’s risk of experiencing IPV. The protective aspects of employment against intimate partner violence for women include their increased access to wider social networks, information and support and the resulting improved confidence and bargaining position in their relationships [10, 11]. Conversely, unemployment prevents women from leaving an abusive relationship and fosters traditional gender roles that put the burden of keeping a violence-free relationship on the shoulders of the woman [12, 13].

These associations are best explained by the resource and relative resource theories. These theories claim that abusive male partners use violence because other resources, including education, employment, job prestige, income or community standing are unavailable to them or fail to obtain the desired response to command dominance and power in their relationships [14]. The relative resource theory posits that economic differences favouring the woman lead to IPV as it challenges established gender norms and may be perceived to threaten the male role as a breadwinner and therefore challenge their masculine identity [15–17].

Cross-sectional studies have established that women’s employment and income can be both a risk and a protective factor for IPV. On the protective side, World Health Organisation’s ‘Multi-country study on women’s health and domestic violence’ and global Demographic and Health Survey (DHS) analyses have found that women’s and their partners’ employment and income tended to be protective against IPV [18–20]. However, the reverse has also been found in societies with rigid gender roles, with relationships where the woman commands more resources than her partner being at greater risk of IPV perpetrated by the male partner [21]. A longitudinal study in Mumbai found that a women’s joint control over her husband’s income and her financial inclusion as indicated by bank ownership appear to reduce her risk for IPV, whereas her income generation or control over her own income do have a null effect [22]. Other research has found that change in employment can be protective or risky for partner violence. A longitudinal study of married women in Bangalore found that newly-employed women faced higher odds of violence and that husbands who lost their job during the study were more likely to abuse their partners [21].

Violence may also have later effects on economic earnings. A longitudinal analysis of more than 200 pregnant adolescents in the United States found that IPV has negative effects on economic capacity even many years after the violence occurs [23]. This suggests a bidirectional relationship between economic status and IPV.

Little research in sub-Saharan Africa has explored variations by context (urban versus rural) and disaggregation by age with regard to IPV and economic empowerment, despite existing knowledge that gender norms that are strongly associated with IPV vary across urban and rural sites and age groups [24, 25]. A cross-sectional study in an urban and rural site in Tanzania only found an association between women earning income and higher levels of IPV in the urban site, hypothesising that the lack of association in the rural site might be mediated by reduced conflict in the household due to the availability of money [26]. Also, little is known about the effect of men’s access to economic resources and the link to IPV perpetration in its different forms as most studies only look at women’s indicators of economic empowerment in relation to men and draw data on men from women only.

This study seeks to answer these gaps using couples data from the Demographic Health Surveys (DHS) in sub-Saharan Africa to investigate the relationship between economic empowerment and IPV and whether this association varies between urban and rural sites and by women’s age.

## Methods

### Demographic health surveys (DHS)

This study utilises couples data from the DHS, spanning survey data collected from 2001 to 2015 in multiple countries. DHS surveys were implemented by respective national institutions and ORC Macro International Inc. with financial support from the US Agency for International Development. The DHS surveys are standardised across countries and years, at the individual and at the household (couples) level, with sample sizes between 5000 and 15,000 households. They are based on probabilistic samples originating from multi-stage cluster sampling and are stratified by rural and urban areas for different regions of the countries. The surveys are conducted on a sample of female respondents aged 15 to 49 years, and increasingly, men aged 15 to 59 years are being sampled and interviewed. Couples datasets merge data from women and their partners who live within the same household. Information on IPV has been collected since 2000 and the DHS programme has developed a standard module and methodology for the collection of data on domestic violence.

Up to 2017, 21 countries in sub-Saharan Africa have DHS surveys that contain information on experience with IPV: Burkina Faso, Cameroon, Congo Democratic Republic, Cote d’Ivore, Gabon, Gambia, Ghana, Kenya, Liberia, Malawi, Mali, Mozambique, Namibia, Nigeria, Rwanda, South Africa, Tanzania, Togo, Uganda, Zambia and Zimbabwe. For the countries in sub-Saharan Africa that implemented the IPV module, only one randomly selected woman per household was eligible to participate to maintain confidentiality and ensure the woman’s security when answering the question on the experience of domestic violence. The development of the domestic violence module was guided by available research on valid and reliable measurement of domestic violence and by World Health Organization (2001) guidelines on the safety and ethical aspects of collecting information on violence against women. The module asking about IPV is based on a modified version of the Conflict Tactics Scales [27]. It asks women if they ever experienced a series of behaviourally specific acts of physical or sexual violence by their current or most recent partner. Only women who have ever lived with a partner are selected to answer the questions about experience with IPV. Women who say yes to a particular item are then asked about the frequency of perpetration in the 12 months preceding the interview. More recent surveys also include questions on emotional abuse.

Interviewers are trained specially to handle the sensitive questions of domestic violence, and they are expected to follow a strict protocol to ensure privacy. Interviewers are instructed to check all the surroundings within hearing distance for the presence of others. Only children young enough to not understand the questions can be present. The interviews are not allowed to proceed if privacy is not ensured, and the interview is terminated if someone enters the interview area [27].

The ICF International Institutional Review Board (IRB) has reviewed and approved procedures and questionnaires for standard DHS surveys. Additionally, country-specific DHS survey protocols are reviewed by the ICF IRB and typically by an ethics committee in the host country. This secondary data analysis has also received ethical approval by the London School of Hygiene and Tropical Medicine ethical review board.

### Measures

The Domestic violence module is based on a modified version of the Conflict Tactics Scale (CTS) that asks the respondents about their experience of specific acts of physical or sexual intimate partner violence. This allows for comparability across countries [27]. All ever-partnered or currently partnered women are asked if any partner ever did one of the following to them:

Physical violence

1. Pushing, shaking, slapping, throwing something,
2. Twisting an arm,
3. Striking with a fist or something that could cause injury,
4. Kicking or dragging,
5. Attempting to strangle or burn,
6. Threatening with a knife, gun, or other type of weapon, and attacking with a knife, gun, or other type of weapon.

Sexual violence

1. Physically forcing intercourse or any other sexual acts,
2. Forcing her to perform sexual acts with threats or in any other way.

In this paper, we use the indicator measuring women’s experience of physical and/or sexual violence among currently partnered women. Women were considered to have ‘experienced intimate partner violence in the past 12 months’ if they reported experiencing at least one physically or sexually violent act in the last year and ‘ever experienced intimate partner violence’ if they reported experiencing any act in their lifetime.

We measured economic empowerment using the following indicators: household wealth of the household the couple was residing in, women’s and their partners’ educational level, employment and earnings, individually as reported by the woman and her partner and in relation to each other, and income related decision-making. Household wealth is a standardised measure of the relative wealth of a household and is measured using the wealth index provided in the DHS. The index is based on household information regarding assets, type of flooring, water supply, electricity, and the ownership of durable goods. It does not include household members’ education or employment status and divides households into poor, middle and rich levels [28]. Education is captured through a categorical variable: no formal education, elementary, secondary and post-secondary education. Employment status is self-reported i.e. if the individual reports being employed, including in the 12 months prior to the interview. Relative income is measured by whether the woman earns more, the same or less than her husband. Decision-making on income is collected through the women’s survey, and based on the woman’s report on whether she decides on her own, with her partner or with someone else; if only her partner, or someone else, decides for her; or if she does not have any earnings of her own. The definition of a cluster as urban or rural is made according to the definition used in each country [29].

### Analysis

Our analysis utilises merged couples data from 25 surveys of the DHS conducted in 15 sub-Saharan African countries where women and their partners participated in the domestic violence module of the DHS. The sample size is 70,993 women aged 15 to 49 years. We used weights to adjust for sampling and domestic violence survey participation.

To analyse the data, we explored descriptive statistics and possible associations between sexual and/or physical IPV in the last 12 months and the seven economic empowerment factors described above by conducting cross-tabulations and chi-square statistics to assess whether an association exists between the outcome and the explanatory variable. Next, we estimated odds ratios using multivariate logistic regression, adjusting for other economic factors in the model as well as age, urban–rural and marital status and country to assess whether the established associations were the true effect or mediated by other variables in the regression. Because these data are cross-sectional, none of the associations can be interpreted as suggesting causality. A p-value below 0.05 indicates statistical significance. All data were analysed using STATA 14. Sensitivity analyses were conducted running the regressions without controlling for country fixed effects as well.

Ethical approval for the secondary analysis was received from the London School of Hygiene and Tropical Medicine Ethical Approval Committee, approval number 14402.

## Results

Among all women included in the couples data, the lifetime prevalence of IPV was 30%, with 20% of women reporting past year IPV. The prevalence rates in individual countries and for physical and sexual violence separately are listed in Table 1. While lifetime and past year prevalence rates notably differ in some countries, e.g. Kenya and Togo, they are close in most sub-Saharan countries, e.g. Sao Tome and Principe. Across the included countries and surveys, as outlined in Table 2, most women lived in households considered to be in the lowest tertile of the wealth measurement (41%) except in urban sites where more households fell into the wealthy tertile (85%). The majority of women had primary education (41%) and 63 percent were currently working, a rate that was lower among women aged 15 to 24 (51%). Among men, the majority also had primary education (39%) and 92 percent were currently working, and the rate did not differ much across rural and urban sites or age groups. In the majority of cases the woman had no income of her own (54%), which was more often the case in rural areas (58%) and among young women (65%) and in 89 percent of the couples the men earned more than the woman, even though he only had a higher education in 29 percent of the cases and both were working in 63 percent of the couples interviewed. A table detailing descriptive statistics by country are displaced in Additional file 1.

**Table 1:** Prevalence of different forms of intimate partner violence across Sub-Saharan countries

	Sexual and/or physical	Sexual	Physical	Physical and or sexual in the last 12 months	Sexual—last 12 months	Physical last 12 months
Burkina Faso 2010	12%	1%	11%	10%	1%	10%
Chad 2014/15	30%	10%	27%	19%	7%	17%
Cote D'Ivoire 2011/12	25%	6%	24%	23%	5%	22%
Ghana 2008	21%	6%	19%	19%	5%	17%
Kenya 2003	41%	14%	37%	29%	12%	25%
Kenya 2008/09	34%	13%	32%	30%	12%	28%
Kenya 2014	37%	11%	35%	27%	9%	24%
Liberia 2007	38%	9%	35%	36%	9%	33%
Mali 2006	21%	4%	20%	18%	3%	17%
Mali 2012/13	35%	13%	31%	28%	12%	22%
Malawi 2004/05	26%	13%	19%	20%	11%	13%
Malawi 2010	27%	15%	20%	22%	14%	14%
Malawi 2015/16	32%	17%	23%	24%	16%	15%
Nigeria 2008	17%	4%	16%	14%	3%	13%
Nigeria 2013	15%	4%	13%	10%	3%	9%
Rwanda 2005	36%	12%	33%	25%	10%	21%
Rwanda 2010	54%	14%	54%	49%	13%	49%
Rwanda 2014/15	32%	9%	29%	22%	8%	19%
Sao Paolo and Principe 2008/09	29%	9%	29%	29%	8%	28%
Tanzania 2009/10	35%	13%	32%	33%	12%	29%
Tanzania 2015/16	40%	12%	37%	33%	11%	29%
Togo 2013/14	24%	7%	22%	14%	5%	12%
Zambia 2007	48%	16%	45%	43%	15%	39%
Zambia 2013/14	41%	15%	37%	28%	13%	22%
Zimbabwe 2005/06	37%	14%	31%	33%	13%	28%
Zimbabwe 2010	35%	16%	29%	30%	15%	22%
Zimbabwe 2015	33%	9%	30%	20%	7%	16%
Total	30%	10%	27%	23%	9%	20%
N	20,376	6870	18,247	16,138	6113	13,718

**Table 2:** Sample characteristics

	All		Urban–rural		Woman age		
	N	Total (%)	Urban (%)	Rural (%)	15–24 (%)	25–34 (%)	35–49 (%)
*Household wealth index*							
Middle	13,679	20	9	23	20	19	20
Poorer	29,152	41	5	55	47	39	39
Richer	25,887	39	85	22	33	42	41
*Woman's secondary education*							
Primary	28,394	41	32	44	42	40	41
None	22,756	34	17	40	33	31	38
Secondary	15,359	22	41	15	25	24	18
Higher	2201	3	9	1	1	4	4
*Woman currently working*							
No	25,922	37	39	36	49	35	29
Yes	42,587	63	61	64	51	65	71
Man's age							
15–34	30,901	42	40	43	84	43	2
35–49	31,898	48	51	47	15	54	70
50 +	5919	10	9	10	1	3	28
*Man's secondary education*							
Primary	27,027	39	26	44	38	38	41
None	16,763	24	11	29	25	23	26
Secondary	20,311	29	46	23	33	31	24
Higher	4612	7	17	3	4	8	8
*Man currently working*							
No	5811	8	5	10	9	7	9
Yes	62,835	92	95	90	91	93	91
*Does she have any say in how her earnings are spent?*							
Yes	26,255	40	52	35	29	42	46
No	4575	7	5	7	6	7	7
No income of her own	37,888	54	43	58	65	51	47
*Who earns more*							
Same	4402	6	6	6	4	7	8
She	2759	4	7	3	2	4	6
He	61,477	89	87	90	94	89	86
*Who has the higher level of education*							
He	19,882	29	32	28	28	29	31
Same	41,718	61	56	62	62	60	60
She	7118	10	12	9	10	11	9
*Who is currently working*							
Both	42,922	63	60	64	52	65	70
He	22,877	33	37	31	43	31	25
She	2919	4	3	5	4	4	5
*Income in cash*							
Not paid in cash	15,452	32	12	39	38	30	30
Paid in cash	31,024	68	88	61	62	70	70
*Woman age*							
15–24	19,417	27	24	29			
25–34	30,974	43	47	42			
35–49	18,327	30	29	30			
*Urban–rural*							
Urban	19,064	28			24	30	27
Rural	49,654	72			76	70	73

Table 3 illustrates that household wealth plays a more prominent role for IPV in rural than in urban sites. There is a significant decrease in IPV in both the unadjusted and adjusted odds ratios in rural areas if the household is richer, compared to being in the middle tertile of the wealth measurement. The same association does not hold in the urban areas. IPV also varied by women’s age: unadjusted odds ratios show that older women (women aged 25 to 34 years compared to women below 25 years) reported less IPV in urban areas but increased violence in rural areas. Having a partner older than 24 years and educational attainment higher than primary level for the woman and her partner decreased IPV across both urban and rural sites. While women being employed in the last 12 months increased the likelihood of reporting IPV across urban and rural sites, men’s employment in the last 12 months increased violence only in rural sites.

**Table 3:** Unadjusted and adjusted odds ratios* of last year physical and/or sexual intimate partner violence and women's and men’s individual indicators of economic empowerment among women aged 15 to 49, nationally (n = 68,426) and in urban (n = 18,981) and rural (n = 49,445) sites

	All		All		Urban		Urban		Rural		Rural	
	OR	95 CI	AOR	95 CI	OR	95 CI	AOR	95 CI	OR	95 CI	AOR	95% CI
*Household wealth (Ref: Middle)*												
Poorer	0.91**	[0.85,0.97]	0.95	[0.89,1.01]	1.07	[0.87,1.32]	1.15	[0.93,1.42]	0.90**	[0.84,0.96]	0.96	[0.90,1.03]
Richer	0.84***	[0.78,0.90]	0.89**	[0.83,0.96]	0.84*	[0.72,0.98]	0.92	[0.78,1.09]	0.87***	[0.80,0.95]	0.88**	[0.81,0.95]
*Woman’s Age* (ref: 15–24)												
25–34	1.04	[0.98,1.10]	1.08*	[1.01,1.15]	0.85*	[0.76,0.96]	0.94	[0.81,1.10]	1.12***	[1.05,1.19]	1.12***	[1.05,1.20]
35–49	0.89***	[0.83,0.95]	0.97	[0.88,1.06]	0.67***	[0.57,0.78]	0.77*	[0.62,0.96]	0.98	[0.91,1.05]	1.04	[0.95,1.15]
*Men’s age* (ref: 15–24)												
25–34	0.77***	[0.74,0.81]	0.84***	[0.79,0.90]	0.67***	[0.60,0.74]	0.79***	[0.69,0.91]	0.82***	[0.78,0.87]	0.86***	[0.81,0.92]
35–49	0.66***	[0.60,0.73]	0.77***	[0.69,0.86]	0.59***	[0.48,0.73]	0.70**	[0.55,0.89]	0.69***	[0.62,0.76]	0.79***	[0.70,0.89]
*Woman’s educational level (Ref: Primary)*												
None	0.51***	[0.48,0.54]	0.75***	[0.70,0.81]	0.51***	[0.44,0.60]	0.62***	[0.53,0.73]	0.51***	[0.47,0.54]	0.78***	[0.72,0.84]
Secondary	0.73***	[0.68,0.77]	0.85***	[0.80,0.92]	0.71***	[0.64,0.79]	0.83**	[0.73,0.94]	0.76***	[0.71,0.82]	0.85***	[0.78,0.93]
Higher	0.31***	[0.26,0.37]	0.49***	[0.40,0.60]	0.32***	[0.26,0.40]	0.52***	[0.40,0.67]	0.31***	[0.23,0.43]	0.47***	[0.33,0.66]
*Man’s educational level (Ref: Primary)*												
None	0.51***	[0.47,0.54]	0.80***	[0.74,0.86]	0.58***	[0.48,0.69]	0.91	[0.76,1.10]	0.50***	[0.46,0.53]	0.77***	[0.71,0.84]
Secondary	0.84***	[0.79,0.88]	0.93*	[0.87,0.99]	0.83**	[0.74,0.93]	0.85*	[0.75,0.96]	0.86***	[0.81,0.92]	0.95	[0.88,1.02]
Higher	0.43***	[0.39,0.49]	0.65***	[0.57,0.74]	0.41***	[0.35,0.49]	0.54***	[0.44,0.66]	0.51***	[0.43,0.61]	0.79*	[0.66,0.96]
Woman worked in the last 12 months (Ref: no)	1.20***	[1.14,1.27]	1.31***	[1.24,1.38]	1.08	[0.97,1.20]	1.42***	[1.27,1.60]	1.25***	[1.18,1.33]	1.29***	[1.21,1.37]
Man worked in the last 12 months (Ref: no)	0.97	[0.89,1.05]	1.09	[1.00,1.19]	0.79*	[0.64,0.98]	0.87	[0.69,1.09]	1.02	[0.93,1.11]	1.14**	[1.04,1.26]

Exponentiated coefficients; 95% confidence intervals in brackets; * *p* < 0.05, ** *p* < 0.01, *** *p* < 0.001

* Adjusted for all other variables in the table

Household wealth, namely being in the richer segment, did not impact IPV among women aged 15 to 24 but reduced the likelihood of reporting IPV among women aged 25 to 34 and women aged 35 to 49. The male partner working only increased the likelihood of IPV among women aged 35 to 49. Otherwise, no differences emerged in individual indicators for women’s economic empowerment across women’s age groups. Men’s older age and educational attainment higher than primary-level for both the women and the men reduced the likelihood of IPV; whereas women working increased it (illustrated in Table 4).

**Table 4:** Unadjusted and adjusted odds ratios* of last year physical and/or sexual intimate partner violence and women's and men’s individual indicators of economic empowerment among women aged 15 to 49, by age groups

	15–24 (n = 19,870)				25–34 (n = 31,793)				35–49 (n = 19,032)			
	OR	95% CI	AOR	95% CI	OR	95% CI	AOR	95% CI	OR	95% CI	AOR	95% CI
*Household Hwealth (Ref: Middle)*												
Poorer	0.84**	[0.75,0.93]	0.95	[0.87,1.04]	0.92	[0.84,1.01]	1.02	[0.95,1.10]	0.97	[0.86,1.09]	1.06	[0.96,1.16]
Richer	0.99	[0.87,1.12]	1.00	[0.91,1.11]	0.79***	[0.72,0.87]	0.86***	[0.80,0.93]	0.80***	[0.70,0.91]	0.83***	[0.75,0.92]
*Men’s age *(ref: 15–24)												
25–34	0.65***	[0.54,0.77]	0.86**	[0.77,0.96]	0.72***	[0.67,0.76]	0.83***	[0.79,0.88]	0.77*	[0.61,0.97]	0.84	[0.69,1.01]
35–49	0.51*	[0.28,0.93]	0.79	[0.51,1.22]	0.56***	[0.46,0.70]	0.85	[0.72,1.00]	0.65***	[0.51,0.83]	0.76**	[0.62,0.93]
*Woman’s educational level (Ref: Primary)*												
None	0.44***	[0.39,0.50]	0.74***	[0.66,0.82]	0.52***	[0.48,0.57]	0.75***	[0.69,0.82]	0.57***	[0.51,0.64]	0.77***	[0.70,0.85]
Secondary	0.81***	[0.73,0.90]	0.82***	[0.75,0.91]	0.70***	[0.64,0.76]	0.89**	[0.82,0.96]	0.64***	[0.56,0.73]	0.82***	[0.73,0.92]
Higher	0.44**	[0.24,0.79]	0.51**	[0.32,0.80]	0.30***	[0.24,0.38]	0.47***	[0.39,0.58]	0.29***	[0.22,0.40]	0.51***	[0.39,0.67]
*Man’s educational level (Ref: Primary)*												
None	0.44***	[0.38,0.51]	0.72***	[0.64,0.81]	0.51***	[0.46,0.56]	0.81***	[0.74,0.89]	0.58***	[0.52,0.65]	0.87*	[0.78,0.97]
Secondary	0.94	[0.85,1.03]	0.95	[0.87,1.04]	0.78***	[0.73,0.85]	0.92*	[0.85,0.98]	0.79***	[0.70,0.89]	0.96	[0.87,1.06]
Higher	0.50***	[0.38,0.65]	0.56***	[0.45,0.69]	0.41***	[0.35,0.48]	0.71***	[0.61,0.81]	0.44***	[0.36,0.53]	0.71***	[0.59,0.85]
Woman worked in the last 12 months (Ref: no)	1.25***	[1.14,1.37]	1.32***	[1.23,1.42]	1.15***	[1.08,1.24]	1.25***	[1.18,1.32]	1.31***	[1.18,1.46]	1.29***	[1.18,1.40]
Man worked in the last 12 months (Ref: no)	0.86*	[0.75,0.99]	0.99	[0.88,1.11]	0.94	[0.83,1.06]	1.08	[0.97,1.19]	1.12	[0.95,1.32]	1.22**	[1.07,1.39]

Exponentiated coefficients; 95% confidence intervals in brackets; * *p* < 0.05, ** *p* < 0.01, *** *p* < 0.001

* Adjusted for all other variables in the table

For relative economic empowerment indicators, women working was a risk factor for increased IPV in both urban and rural settings. When a woman earns more than her husband she has higher odds of reporting violence in urban and rural settings. In urban relationships, the male partner working was protective against violence compared to unemployed men. In rural areas, higher levels of IPV were reported when the woman had a higher level of education than her partner in the unadjusted odds ratios. Apart from that, across urban and rural sites, the likelihood of IPV increased if the woman had no decision-making power over her income. It also increased where the woman earned more than her partner and in rural areas also when had no cash income (see Table 5).

**Table 5:** Unadjusted and adjusted odds ratios* of last year physical and/or sexual intimate partner violence and women's and men’s relative indicators of economic empowerment among women aged 15 to 49 (adjusted for marital status, household wealth, women’s and men’s age in addition to those in the table), nationally (n = 46,396) and by urban (n = 12,260) and rural (n = 34,136)

	ALL		All		Urban		Urban		Rural		Rural	
	OR	CI	AOR	CI	OR	CI	AOR	CI	OR	CI	AOR	CI
*Who decides on her earnings (ref: she)*												
Not She	1.44***	[1.31,1.58]	1.33***	[1.21,1.47]	1.34**	[1.09,1.64]	1.31*	[1.06,1.61]	1.45***	[1.31,1.61]	1.35***	[1.21,1.50]
She has no earnings	1.01	[0.96,1.06]	1.39	[0.87,2.23]	0.95	[0.85,1.05]	0.60	[0.22,1.67]	1.02	[0.96,1.08]	1.85*	[1.10,3.12]
*Who earns more (ref: Same)*												
She	1.34***	[1.17,1.53]	1.41***	[1.23,1.61]	1.58***	[1.24,2.01]	1.52***	[1.19,1.95]	1.26**	[1.07,1.49]	1.35***	[1.14,1.59]
He	0.92	[0.83,1.01]	1.12*	[1.01,1.23]	1.00	[0.82,1.21]	1.09	[0.89,1.34]	0.89*	[0.80,0.99]	1.14*	[1.01,1.27]
*Who has a higher level of education (ref: he)*												
Same	0.96	[0.91,1.01]	0.98	[0.92,1.04]	1.02	[0.91,1.14]	1.07	[0.92,1.24]	0.93*	[0.87,0.99]	0.96	[0.89,1.03]
She	1.11*	[1.02,1.20]	1.02	[0.93,1.13]	1.11	[0.95,1.30]	1.22*	[1.01,1.48]	1.11*	[1.02,1.22]	0.96	[0.86,1.08]
*Who is currently working (ref: he)*												
Both	0.84***	[0.80,0.88]	0.96	[0.87,1.07]	0.92	[0.82,1.02]	1.12	[0.88,1.43]	0.81***	[0.77,0.87]	0.92	[0.82,1.03]
She	1.08	[0.97,1.20]	0.93	[0.83,1.05]	1.20	[0.89,1.61]	1.10	[0.80,1.51]	1.04	[0.93,1.17]	0.89	[0.79,1.01]
Cash income	0.86***	[0.81,0.92]	1.28	[0.79,2.05]	0.83*	[0.70,0.99]	0.55	[0.20,1.55]	0.89***	[0.83,0.95]	1.70*	[1.00,2.87]

Exponentiated coefficients; 95% confidence intervals in brackets; * *p* < 0.05, ** *p* < 0.01, *** *p* < 0.001

* Adjusted for all other variables in the table

Similarly, as evident in Table 6, few differences emerged in relative economic predictors analysed according to age groups. There were higher levels of IPV reported among women who have no decision-making power over their earnings and those who earn more than their partner. While both individuals working compared to only the male partner working reduced IPV across all age groups, reports of IPV were higher when only the woman was working compared to only the male partner working solely among women aged 15 to 24 years and not in the adjusted analysis. IPV reporting decreased in relationships among women aged 35–49 if only she was currently working. Receiving cash income was associated with decreased IPV only among women aged 25 to 34 years old; however, only women in this age group reported higher IPV if they had a higher level of education.

**Table 6:** Unadjusted and adjusted odds ratios* of last year physical and/or sexual intimate partner violence and women's and men’s relative indicators of economic empowerment among women aged 15 to 49 (adjusted for marital status, household wealth, and urban–rural), by age groups (n = 47,670)

	15–24 (n = 11,174)				25–34 (n = 22,130)				35–49 (n = 14,366)			
	OR	CI	AOR	CI	OR	CI	AOR	CI	OR	CI	AOR	CI
*Who decides on her earnings (ref: she)*												
Not She	1.39***	[1.17,1.65]	1.32***	[1.14,1.53]	1.46***	[1.28,1.65]	1.29***	[1.16,1.43]	1.45***	[1.21,1.73]	1.22**	[1.06,1.40]
She has no earnings	0.96	[0.87,1.07]	1.62	[0.73,3.62]	1.03	[0.96,1.10]	1.21	[0.62,2.36]	0.98	[0.89,1.08]	0.95	[0.44,2.05]
*Who earns more (ref: Same)*												
She	1.43*	[1.04,1.98]	1.60***	[1.23,2.09]	1.49***	[1.23,1.81]	1.40***	[1.20,1.64]	1.20	[0.96,1.50]	1.38***	[1.16,1.65]
He	0.92	[0.76,1.13]	1.33**	[1.11,1.59]	0.94	[0.83,1.07]	1.14*	[1.02,1.28]	0.85	[0.72,1.00]	1.02	[0.89,1.16]
*Who has a higher level of education (ref: he)*												
Same	0.97	[0.88,1.07]	0.93	[0.84,1.02]	0.93	[0.86,1.01]	0.97	[0.90,1.04]	0.97	[0.88,1.08]	0.97	[0.89,1.07]
She	1.02	[0.88,1.18]	0.92	[0.78,1.07]	1.15*	[1.02,1.28]	1.02	[0.91,1.13]	1.11	[0.95,1.30]	1.03	[0.89,1.18]
*Who is currently working (ref: he)*												
Both	0.82***	[0.75,0.90]	0.98	[0.84,1.13]	0.86***	[0.80,0.92]	0.95	[0.85,1.07]	0.78***	[0.70,0.88]	0.99	[0.84,1.17]
She	1.30**	[1.07,1.59]	1.14	[0.97,1.35]	1.05	[0.89,1.23]	0.87	[0.76,1.00]	0.95	[0.77,1.19]	0.81*	[0.69,0.96]
Cash income	0.89	[0.89,0.89]	1.46	[0.65,3.28]	0.85***	[0.79,0.93]	1.14	[0.58,2.22]	0.87	[0.87,0.87]	0.92	[0.43,1.97]

Exponentiated coefficients; 95% confidence intervals in brackets; * *p* < 0.05, ** *p* < 0.01, *** *p* < 0.001

* Adjusted for all other variables in the table

## Discussion

Our analysis of DHS data across 15 countries provides patterns of associations between IPV and economic indicators across urban versus rural sites and across age groups. Physical and/or sexual IPV is highly prevalent across sub-Saharan Africa, with 20% of women reporting it in the past year. This study shows that IPV was associated with a variety of factors indicative of women’s economic empowerment. While most of the factors operated similarly in urban and rural settings and across age groups, notable differences emerged. For example, household wealth and women’s age was associated with reduced likelihood of IPV in rural areas while men being the only partner employed reduced IPV in urban areas. Factors that are indicative of relative economic empowerment (e.g., relative educational level or relative earnings) showed few differences across urban versus rural sites and age groups in respect to IPV.

The study findings do not support the resource theory claim that women’s employment reduces IPV by increasing their social network, information and support. However, the increase in IPV if the woman works supports the relative resource theory argument that women’s economic success may threaten existing gender roles. This is further supported by the significant increases in IPV found in this study if the woman is earning more than her partner compared to both earning the same. This study provides further support for this, by showing that in rural areas where gender norms are more rigid and higher pressure is put on men to be the main breadwinner [25], women who have a higher education also have a higher risk for IPV. It is indicative of broader unequal power relations within relationships, where the man is better educated or has a higher income or is perceived as a transgression of gender norms where the woman is more highly educated or the only breadwinner. In settings where women do not commonly work outside the home or where this is reflected to stand for the inability of the partner to provide for his family, women’s entry into employment and higher income might increase marital tensions as men might not immediately recognise the benefits of additional household income [22, 30]. Marital tensions might also increase because increased financial independence might allow the woman to push for change within a relationship and increase the risk of her leaving her partner, which might be perceived as threatening to his status [16–18, 31, 32].

Our analysis also shows that IPV is associated with economic stress for the family, with greater household wealth and higher levels of education (as a proxy for higher earning potential) associated with lower levels of IPV [34]. This is across urban and rural sites and most age groups. In addition, male partner’s employment appears to be a protective factor in urban sites and among women under 25 years of age. Household wealth also only decreased IPV among women aged 25 and older, not among younger women (< 25 years). Others have hypothesised that the association between relative financial contribution and IPV might be as much linked to poverty as the partner’s failure to fulfil the expected male role of provider, as to the woman’s economic position and her perceived transgression of gender roles per se [33, 34].

Overall, the findings of our analysis highlight that despite there being no major differences in terms of women’s economic empowerment indicators across urban versus rural sites and across age groups, some differences are important. Future analyses should investigate further the mechanisms linking different indicators to women’s experience of IPV and whether they differ across contexts and age groups. Investigating regional patterns or taking into account cultural, political or other relevant contextual factors will be a next step of this research. Sub-Saharan African economies are faced with an increased level of informal employment that challenges existing employment patterns among men and increases women’s labour force participation, which influences gender roles and IPV levels [31], but this effect has not yet been sufficiently explored. Our findings also show that relative indicators of economic empowerment are also important and that women’s advancements in terms of employment or education might lead to a backlash if their partner does not enjoy the same level of education or employment. Future programmes therefore need to find ways to involve women’s partners if we seek to have a lasting reduction in levels of IPV.

It is important to mention that our analysis has some key limitations. We used couples data to base our findings on reports directly from men and women, however it means that the analysis was restricted to women who currently have a partner who agreed to be interviewed as well. This excludes women in more unstable relationships or women who have recently separated from their partner. The study is cross-sectional in nature, thereby not allowing us to make any claims regarding causality, and it is well established that factors around economic empowerment of women can both be a cause as well as a consequence of IPV [18]. Using the DHS allowed the inclusion of multiple countries in sub-Saharan Africa into this analysis, with the caveat that the number of factors related to women’s economic empowerment was limited, especially the more latent construct regarding women’s and men’s perception about their contributions, their mental health that is an important mediating factor in this analysis and their general attitudes towards gender norms and women’s empowerment. While this analysis has focused on physical and/or sexual  IPV only, future studies should also investigate economic and emotional abuse [35].

## Conclusion

This paper highlights the complex nature of women’s economic empowerment and its association with IPV. It further showed that in order to economically empower women, we need to work with both men and women to address gender roles and livelihood opportunities for both sexes. It highlights the need for future research studies using couples data [36]. For intervention and programme development, we need to understand the target population well and not assume similar effect and mechanisms across broad population groups and design interventions and programmes accordingly. Couples interventions have emerged to be promising investments into the global effort to reduce IPV, with economic decision-making and communication between the couples playing a crucial role. The findings of this study provide further insights into aspects that need to be considered when designing programmes to take into account the differences between urban and rural as well as couples across different age groups.

## Supplementary Information

**Additional file 1.** A table detailing descriptive statistics by country are displaced in Additional file.

## Data Availability

Access to the publicly available Demographic and Health Survey data can be requested under https://dhsprogram.com/data/available-datasets.cfm

## Abbreviations

DHS: Demographic and Health Surveys
IPV: Intimate partner violence
