# Supplementary material for: Economic empowerment and intimate partner violence: a secondary data analysis of the cross-sectional Demographic Health Surveys in Sub-Saharan Africa
Source: BMC Womens Health. 2021 Jun 12;21:241. doi: 10.1186/s12905-021-01363-9 (PMC8199805; doi:10.1186/s12905-021-01363-9)
Supplement: Supplementary file 1 — Additional file 1. A table detailing descriptive statistics by country are displaced in Additional file. [file 12905_2021_1363_MOESM1_ESM.docx]

**Supplementary file - Response to BMC Public Health Revisions**

**Official response letter stating that BMC Public Health could not find enough reviewers and therefore rejected the manuscript after two months.**

PUBH-D-20-00507
Economic empowerment and intimate partner violence: an analysis of Demographic Health Surveys in Sub-Saharan Africa
Heidi Stöckl; Anushe Hassan, MSc; Meghna Ranganathan, PhD; Abigail Hatcher, PhD
BMC Public Health

Dear Dr Stöckl,

Thank you for considering BMC Public Health for your manuscript. I am sorry to inform you that despite much effort we have been unable to obtain appropriate referees for your manuscript in a timely manner. Because we do not feel able to provide you with an editorial decision within a reasonable timeframe we believe it is unfair to hold on to the manuscript and we are closing your file, so that you might have the opportunity to submit elsewhere.

We understand that this decision may cause frustration to you and your co-authors and we sincerely apologise for this inconvenience.

We would like to assure you that we are reviewing the circumstances which led to your manuscript being held for what we consider an unacceptably long timeframe, in order to prevent recurrence. We do hope this will not prevent you considering submission to BMC journals in the future.

I wish you every success with your research and hope that you will consider us again in the future.

Best wishes,

Kevin Zhu
BMC Public Health
[https://bmcpublichealth.biomedcentral.com/](https://protect-eu.mimecast.com/s/u8QRC5LPGC8V1EINfiwj?domain=bmcpublichealth.biomedcentral.com)


Editor Comments:

**Detailed response to reviewer 1:**

Reviewer 1: Abstract
The objective is not clear. Statistics employed was not stated. Weak link between the results and the conclusion.

***We have rewritten the abstract and made several changes to it, including making the results clearer regarding the direction of the associations. We have also rewritten the conclusion section.***

Background.
This section, especially the introductory part is written in such a way it is difficult to understand the focus of the paper. I suggest that this part should not be too 'complex' from the beginning.

***We have added explanations to the background that will make the background more accessible to readers outside the field. We still believe that the background should state the key existing theories on intimate partner violence and economic factors predicting it and explain to the reader the complex and divergent findings that other studies have found.***

Method
It was stated that the data was collected between 2001 and 2015. We are in year 2020, thus, the data was collected between 19 and 5 years ago. How recent the data is a concern that must be addressed. What years was used for this study? Are they the most recent?

***The first Demographic and Health Survey (DHS) in sub-Saharan Africa that collected data on IPV has been conducted in 2001. Each country that has suitable data available – couple data in addition to data collected on IPV – has a different time of data collection as this is not a multi-country survey but an ongoing survey conducted in different countries in different years. Time between data collection and data release is up to 2 years. Instead of deciding on an arbitrary cut off in terms of years, we have decided to use all available data, even if collected in the same country, as not the same women were interviewed in each country. We decided to rather use a wide spread of countries than using only the most recent data in a selected number of countries. In addition, as prevalence rates of IPV did not change significantly between countries over the years, in case more than one survey was available, there is little reason to believe that the risk factors also changed dramatically. We therefore also merged the available survey data in one country across different years.***

Measure- Did you use 'ever-partnered women' or 'currently partnered women' or both? The reason why I am asking is because most explanatory variables are current, and they ought to be matched with recent events (in the last 12 months).

***We used currently partnered women only, as we could otherwise not use couple data, as outlined in the methods section. We have clarified this again in the measurement part.***

Also, its good you extend the economic empowerment to the men and not only the women. However, some variables like household wealth are not necessarily for the couple or individuals, its for the whole household. In some societies in Africa, where extended family system is being practiced, the man and/or the woman may contribute nothing or little to the household wealth. The house may belong to parents, sibling or in-laws. Also, some variables like education are for individuals and may be difficult to measure for households or couples. Therefore, clarity is needed on measurement of economic empowerment in this study. Please confirm categorization of household wealth in DHS surveys. Wealth status was categorized into quintile (five categories) in the Individual data sets.

***We have clarified that the household wealth refers to the household the couple resides in. The woman and her partners education and employment level was reported by them individually and this is stated as well. The household wealth index is also explained in detail, with a reference to the DHS for further detail if required.***

The definition of urban and rural is lacking in context of this work (or in DHS surveys)? And how feasible to generalized this definition for a multi-country study?

***As outlined, the DHS is not a multi-country dataset. The definition of urban and rural in the DHS follows definitions used in each country. This was clarified using a reference to the relevant DHS publication.***

Justification for the use of chi-square and multivariate logistic regression was not stated.

***We have added the justification to the text.***

Analysis
The study used 25 surveys conducted in 15 countries. How? Why? (For example, how did you arrive at 25 surveys? Why did you use data for 15 countries? why not less?

***We have now stated that we have merged the existing couple data from all available surveys to utilize it for our analysis, which are 25 from 15 countries. As stated before, we used all available data without making an arbitrary cut off. As now stated early in the introduction, prevalence rates in urban and rural areas differ even within the same country. As our study’s aim is to investigate if economic empowerment factors have the same effect on the increase or reduction of IPV across urban and rural areas and across different age group, a separation of this sort was most feasible. Combining data from various countries follows the tradition of many other analyses of this kind, e.g. Cools and Kotsadam 2017 or Ellsberg et al 2008***

In this manuscript, the study area, sub-Saharan Africa (SSA) was presented as if it's a country. SSA has varying economic, social and reproductive health indicators. Even, these indicators vary within countries and not only across countries. The discussion was not robust enough as very little is said about similarities and differences across countries and between rural and urban areas.

***The study’s aim was not to investigate differences across countries, but to explore if the same factors around economic empowerment lead to increases or reduction in IPV in urban and rural areas and across different age groups. We have highlighted that reasons for the expected difference in urban and rural areas may be due to gender norms often being more rigid in rural areas and therefore higher pressure is put on men to be the main breadwinner in their household.***

Apart from descriptive statistics (frequencies and percentages), inferential statistics mentioned in the method are not discussed. I suggest the authors should indicate statistical outputs for chi-square and multivariate logistic regression, with their interpretation/discussion in the work.

***The manuscript tried to avoid duplication by providing all numbers with all and detailed statistical outputs in the tables, while describing the findings in the results section in words to ensure that both readers who prefer numbers and readers who prefer text have the format they prefer available. While not stating statistical outputs in terms of numbers, the text clearly refers to associations that were statistically significant or not.***

Conclusion

The conclusion should be result driven. From the title, I don't think the study is only on women, thus the conclusion section should also accommodate men.

***We have also included a section discussing the importance of also working with men in IPV prevention programmes into the discussion, as it is visible from our results that relative indicators of economic empowerment are important. The conclusion section also refers to it.***
